# Low and High Expressing Alleles of the *LMNA* Gene: Implications for Laminopathy Disease Development

**DOI:** 10.1371/journal.pone.0025472

**Published:** 2011-09-29

**Authors:** Sofía Rodríguez, Maria Eriksson

**Affiliations:** Department of Biosciences and Nutrition, Center for Biosciences, Karolinska Institutet, Huddinge, Sweden; Institut Jacques Monod, France

## Abstract

Today, there are at least a dozen different genetic disorders caused by mutations within the *LMNA* gene, and collectively, they are named laminopathies. Interestingly, the same mutation can cause phenotypes with different severities or even different disorders and might, in some cases, be asymptomatic. We hypothesized that one possible contributing mechanism for this phenotypic variability could be the existence of high and low expressing alleles in the *LMNA* locus. To investigate this hypothesis, we developed an allele-specific absolute quantification method for lamin A and lamin C transcripts using the polymorphic rs4641^C/T^ *LMNA* coding SNP. The contribution of each allele to the total transcript level was investigated in nine informative human primary dermal fibroblast cultures from Hutchinson-Gilford progeria syndrome (HGPS) and unaffected controls. Our results show differential expression of the two alleles. The C allele is more frequently expressed and accounts for ∼70% of the lamin A and lamin C transcripts. Analysis of samples from six patients with Hutchinson-Gilford progeria syndrome showed that the c.1824C>T, p.G608G mutation is located in both the C and the T allele, which might account for the variability in phenotype seen among HGPS patients. Our method should be useful for further studies of human samples with mutations in the *LMNA* gene and to increase the understanding of the link between genotype and phenotype in laminopathies.

## Introduction

Mutations in the *LMNA* gene are responsible for a broad range of phenotypically distinct disorders called laminopathies. The diseases are often subclassified into lipodystrophies, muscular dystrophies, neuropathies, and premature aging syndromes [1]. The *LMNA* gene is located on chromosome 1q21.1–21.2 and comprises 12 exons [2], [3]. It encodes five A-type lamins (A, AΔ10, AΔ150, C, and C2) produced by alternative splicing. Lamin A and lamin C correspond to the 2 major lamin isoforms, and lamin C has the highest expressed transcript [4]. Lamin A is encoded by exons 1–12, while lamin C is encoded by exons 1–10 and possesses five unique basic amino acid residues at its C-terminus. Lamin C2 is identical to Lamin C, except that it is encoded by an alternative exon 1 located in the first intron of the *LMNA* gene. Lamin C2 has only been described in mice and human germ cells [5], [6]. Lamin AΔ10 is identical to lamin A but has no exon 10 and has been described in cancer cells [7]. Lamin AΔ150 is the isoform that has been associated with HGPS and is only expressed at very low levels in normal cells [1], [4], [8].

Different mutations in the *LMNA* gene can cause the same type of disorder, but more interestingly, the same base substitution can cause different disorders. One example is the c.1824C>T, p.G608G mutation. This mutation in exon 11 of the *LMNA* gene causes the segmental premature aging disease HGPS or the more severe lethal skin disease restrictive dermopathy [9]. Another example is the c.1930C>T, p.R644C mutation responsible for a very high phenotypic variability between individuals [10]. *LMNA* mutations sometimes also result in disorders that present several overlapping and different phenotypic syndromes of varying severities, e.g., Emery-Dreifuss muscular dystrophy, lipodystrophy and cardiac conduction abnormalities [11]. In some cases, individuals with *LMNA* mutations are asymptomatic [10], [12]. This variability provides evidence for mechanisms, in addition to the specific *LMNA* mutation, that would influence the phenotypic outcome, and it highlights the importance of the genetic background for the development of an inherited disease.

Differential allelic expression has been estimated to affect 20–50% of genes in humans [13], [14]. Therefore, one possible contributing mechanism for the phenotypic variability in laminopathies could be the existence of low and high expressing alleles in the *LMNA* locus. In this study, we addressed this hypothesis and developed allele-specific absolute quantification assays.

## Analysis and Results

Ethics Statement: All samples were obtained from cell repositories and were therefore exempt from ethical approval from our institutional review board.

Allele-specific absolute quantification assays make use of one of the very few available coding SNPs in the *LMNA* gene, rs4641^C/T^, located in exon 10 and included in the lamin A, lamin AΔ150, Lamin C2 and lamin C transcripts. Even though the assays cross-react with minor isoforms, we have only referred to the major isoforms, the lamin A and C transcripts, throughout the study. Our quantification method used real-time RT-PCR technology with TaqMan probes in combination with allele-specific primers. Absolute quantification was obtained by comparison to a standard curve of plasmids that contained the amplification products for the different allele-specific transcript assays. Our quantitative, allele-specific TaqMan assays measured the relative contribution of each allele to the total amount of the *LMNA* transcripts, lamin A and lamin C. Using this assay in combination with cloned standards for the different transcript assays, we quantified the absolute transcript copy number in 11 primary dermal fibroblast cultures corresponding to 8 different individuals genotyped for rs4641^C/T^: 4 HGPS patients (AG01972B and GM01972B, AG11498 and AG11498A, AG03513E, AG06917A and AG06917B) with a confirmed c.1824C>T, p.G608G mutation and 4 unaffected controls (AG03257, AG03258, AG03512 and AG06299B). All cell cultures except for AG03258 and AG03512 were heterozygous (C/T) for rs4641.

The AG03258 sample was homozygous for the C allele, and sample AG03512 was homozygous for the T allele. These samples were used as controls for the specificity of the allele-specific TaqMan assays.

**Figure 1:**
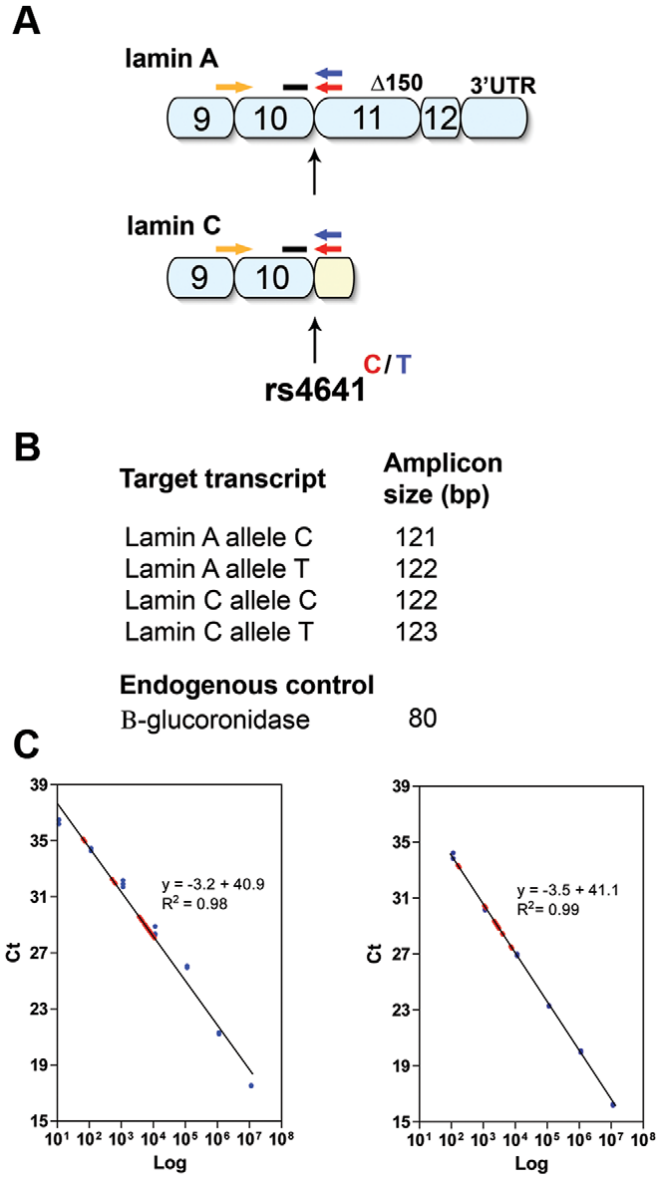
TaqMan assays for allele-specific absolute quantification of the *LMNA* transcripts lamin A and lamin C. (A) Schematic illustration of the location, in the 3′ end of the lamin A and lamin C cDNA, of the primers and probes used in the different assays. The rs4641 SNP is located in the last nucleotide of exon 10, which is present in lamin A and lamin C transcripts. The forward primer, 5′-CAACTCCACTGGGGAAGAAGTG-3′ (orange arrows), and the TaqMan MGB probe, 5′-ATGCGCAAGCTGGTG-3′ (black bars), were the same in all 4 assays. Reverse primers (blue and red arrows) make the assay specific for each allele and transcript by allele-specific 3′-end termination. The reverse primer in the lamin A allele C assay was 5′-GCTGCAGTGGGAGCCG-3′ in the lamin A allele T assay, 5′-TGCTGCAGTGGGAGCCA-3′ in the lamin C allele C assay, 5′-GGCGGCTACCACTCACG-3′, and in the lamin C allele T assay, 5′-CGGCGGCTACCACTCACA-3′. Amplification of the lamin A and lamin C transcripts also results in co-amplification of the lamin AΔ150 and lamin C2, respectively. (B) Target transcripts and sizes of the PCR products of the 4 designed TaqMan assays and the endogenous control. (C) Standard curves of plasmids containing the assay-specific amplicon, estimated absolute copy numbers of transcript lamin C allele C (left) and transcript lamin C allele T (right). A minimum of 5 standards (blue) ranging from 1.1×10^1^ to 1.1×10^7^ estimated copies of plasmids were used. Standards and samples from 11 different human dermal fibroblast cultures (red). Cultures were derived from 4 heterozygote HGPS patients (N = 7), heterozygote controls (N = 2), and homozygote controls (N = 2). Ct: cycle threshold.

**Figure 2:**
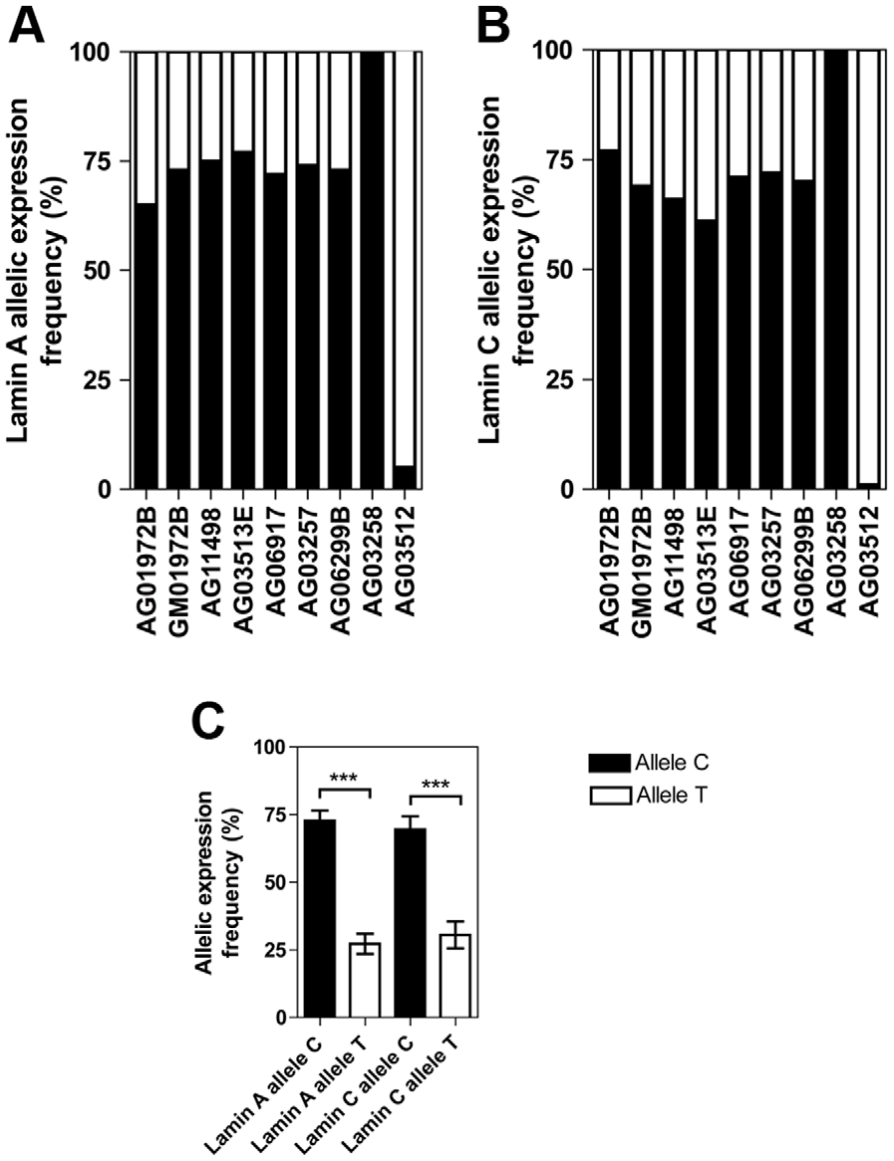
Relative expressed allele frequencies of rs4641 alleles C and T for lamin A and C transcripts. (A, B) Human dermal fibroblast cultures from 7 heterozygous cell cultures and 2 homozygous cell cultures. Estimated absolute copy numbers of the lamin A (A) and lamin C (B) transcripts were normalized to the endogenous control β-glucoronidase. The mean value from different batches of the same cell culture is presented. Mean value of AG11498 and AG11498A is presented as AG11498. Mean value of AG06917A and AG06917B is presented as AG06917. AG01972B and GM01972B correspond to different biopsies from the same patient. Quantification of the homozygous control samples (AG03258 and AG03512) showed only weak amplification with the C assay and no amplification with the T assay in the absence of the respective alleles. (C) Mean and standard deviation of the frequencies of lamin A and lamin C transcripts for each allele across the 7 heterozygous human dermal fibroblast cultures derived from 6 different individuals. Two-tailed unpaired t-test. Asterisks represent significant differences: ***P<0.0001.

Cell culture, RNA isolation and cDNA synthesis were performed as described previously [4]. TaqMan assay primers and minor groove binder (MGB) probe sets for each allele-specific assay were designed using Primer Express software version 2.0 (Applied Biosystems) and Primer 3 [15]. At least one primer per assay was designed to cover an exon-exon boundary, and the location of primer and probes are illustrated in Fig. 1A. Primer and probe sequences for the different assays can be found in the legend to Fig. 1. The different TaqMan assays and the size of the amplification products are shown in Fig. 1B. Preparation of standard curves of plasmids for each assay, real-time PCR conditions, normalization and data analysis were performed as described previously [4]. Representative standard curves for absolute quantification of lamin C transcripts with the C and T allele are shown in Fig. 1C. The absolute values of the transcript copy numbers for each allele-specific assay were normalized to β-glucoronidase. Normalized mean transcript levels from different batches of cell cultures were averaged to a mean value per cell culture. The relative transcript frequencies for each allele and culture were obtained by dividing the amount of transcript from each allele to the total amount of transcripts from both alleles for lamin A and for lamin C (Fig. 2A–B). Finally, the mean transcript frequency for each allele across all informative heterozygous cell cultures was calculated for *LMNA* transcripts lamin A and lamin C (Fig. 2C). The relative transcript frequencies for each allele between informative cell cultures did not vary significantly from each other. For the C allele, they ranged from 0.65 to 0.77 for the lamin A transcript (Fig. 2A) and from 0.61 to 0.77 for the lamin C transcript (Fig. 2B). Accordingly, the transcript frequencies for the T allele ranged from 0.23 to 0.35 and from 0.23 to 0.34 for transcripts lamin A and lamin C, respectively (Fig. 2A–B). The frequency of the transcripts for the homozygous control sample for the C allele was close to 1 for both transcripts (0.9995 for lamin A and 0.9993 for lamin C). A similar situation was observed for the homozygous control sample for the T allele, which had a transcript frequency close to 1 for the lamin A transcript (0.947) and for the lamin C transcript (0.991), indicating high allele-specific specificity for the TaqMan assays (Fig. 2A–B). For the lamin A transcript, the mean proportion of the contribution of each allele to the total amount of transcript across all of the heterozygote samples was 72%±SD 4% for allele C and 27%±SD 4% for allele T (Fig. 2C). For lamin C, the mean proportion of the contribution of each allele to the total amount of transcript across all heterozygote samples was 69%±SD 5% for allele C and 31%±SD 5% for allele T (Fig. 2C). In conclusion, we have high and low expressing alleles for the *LMNA* gene confirmed by similar results in 2 different transcripts. The transcripts from the C allele are more frequent and account for approximately 70% of the total lamin A and lamin C transcripts compared to the lower transcript frequency of approximately 30% from the T allele in all heterozygote samples tested.

We further investigated the implications of whether the localization of an *LMNA* mutation is present on a high or a low expressing allele. For this purpose, we wanted to determine whether the c.1824C>T, p.G608G mutation in classical HGPS showed a preference for a specific allele. The genomic region of the *LMNA* gene that contained the rs4641 SNP in exon 10 and the c.1824C>T mutation in exon 11 was amplified using a high fidelity enzyme and the following primers: 5′-TGACGAGGATGAGGATGGAGA-3′ and 5′-AGTTGCCCAGGAGGTAGGAG-3′. The resulting PCR product was 1039 bp; it was subsequently cloned into the pCR 2.1 Topo cloning vector (Invitrogen), and the inserts were sequenced. Sequencing of the exons 10–11 *LMNA* genomic DNA region showed that the HGPS mutation was either located on the C allele (n = 3; AG03506 (sample originated from the same donor as AG03513), AG06917, and AG11498) or on the T allele (n = 3; AG01972, HGALBV009, and HGALBV011). The samples used for allele-specific transcript quantification (AG03513, AG01972, AG06917 and AG11498), were obtained from 4 skin biopsy donors. All four donors have been described as patients that showed classical features of progeria; however, no additional detailed information about the development of the clinical phenotype is available, making it difficult to relate our results to disease severity (Coriell Cell Repositories, Camden NJ). Two samples, HGALBV009 and HGALBV011, were obtained from the Progeria Research Foundation. In these samples, the mutation was located on the T allele, and the patients from whom they were taken have all of the classical features of Hutchinson-Gilford progeria syndrome; however, these patients were graded as less severe because neither child had heart attacks or strokes by the ages of 14 years, and both grew to above the 95%ile for height of a child with HGPS (yet still below the 3%ile in height for age matched controls) (Dr. Leslie Gordon, 2011, The Progeria Research Foundation Medical and Research Database, Center for Gerontology and Healthcare Research, Brown University, USA). This possible correlation might suggest that if the HGPS mutation is located on the T allele, it can result in a less severe phenotype.

## Discussion

Other studies have related phenotype severity in HGPS to the increased expression of progerin and an increased ratio of progerin to lamin A in the more severe HGPS patients [16], [17]. To test whether the progerin transcript levels were higher when the mutation was located on the C allele compared to the T allele, we reviewed our earlier study in which we quantified the absolute copy number of *LMNA* transcripts lamin A, lamin C and the progerin transcript, lamin AΔ150 [4]. In that study, we found variable but increasing amounts of the progerin transcript with passage number of cells from HGPS patients [4]. However, we did not see any indication that there was a correlation between the expression of progerin and the allelic location of the mutation (data not shown). The progerin/lamin A ratio was 1.0 for AG06917 and 0.9 for AG11498. In both of these cell lines, the mutation is located in the C allele. For sample AG01972, in which the c.1824C>T mutation is located on the less frequently expressed T allele, the progerin/lamin A ratio was slightly lower, 0.8 (data not shown). This observation is interesting, but further investigations on additional samples and on specific mechanisms are needed. An important focus for future studies will be to determine if there is a correlation between the allele-specific location of different *LMNA* mutations and the severity and development of the clinical phenotype.

There have been other explanations raised to try to explain the phenotypic variance among the laminopathies [10], [18]. These explanations include digenic inheritance, with a second mutation that still remains to be identified in the *LMNA* gene, the *ZMPSTE24* gene or any other gene. There are also reports suggesting that some of the laminopathies are better described as intermediate syndromes because the patients have additional clinical features that are less related to laminopathies [10], [18].

In this study, we have developed an allele-specific assay for the *LMNA* gene and showed that there is differential allelic expression of this gene. Whether the different expression of the two alleles might be caused by the rs4641 variant or a second variant, possibly in the promotor or enhancer region, located in the same haplotype block, remains to be investigated. Our allele-specific absolute quantification method is useful for further studies on laminopathies and presents the possibility of investigating whether differential allelic expression can explain the very high phenotype variability that is associated with these diseases and whether this can be used to predict phenotypic severity.
